# Association of Pain, Severe Pain, and Multisite Pain with the Level of Physical Activity and Sedentary Behavior in Severely Obese Adults: Baseline Data from the DieTBra Trial

**DOI:** 10.3390/ijerph17124478

**Published:** 2020-06-22

**Authors:** Carolina Rodrigues Mendonça, Matias Noll, Ana Paula dos Santos Rodrigues, Priscila Valverde de Oliveira Vitorino, Márcio de Almeida Mendes, Erika Aparecida Silveira

**Affiliations:** 1 Postgraduate Program in Health Sciences, Faculdade de Medicina, Universidade Federal de Goiás, Goiás 74605-050, Brazil; anapsr@gmail.com (A.P.d.S.R.); erikasil@terra.com.br (E.A.S.); 2 Instituto Federal Goiano (IF Goiano), Goiás 74270-040, Brazil; matias.noll@ifgoiano.edu.br; 3 Postgraduate Program in Health Sciences, Professor of the School of Social Sciences and Health, Pontifical Catholic University of Goiás, Goiânia, Goiás 74605-010, Brazil; fisioprivitorino@gmail.com; 4 School of Physical Education, Federal University of Pelotas, RS 96020-220, Brazil; marciopenha.esef@yahoo.com.br

**Keywords:** musculoskeletal pain, morbid obesity, arthritis, muscle relaxants, central, sedentary lifestyle

## Abstract

The study aimed to assess the prevalence of pain, severe pain, and pain in four or more regions associated with physical activity and sedentary behavior, as well as other associated factors in severely obese adults (Body Mass Index ≥ 35 kg/m^2^). Baseline data from the DieTBra Trial were analyzed. The outcome variables were pain (yes/no) and pain in four or more sites (yes/no), as identified by the Brazilian version of the Nordic Musculoskeletal Questionnaire, along with the presence of severe pain (yes/no), identified based on the Numerical Pain Rating Scale (≥8). The main independent variables were moderate to vigorous physical activity (MVPA), light physical activity, and sedentary behavior, assessed by triaxial accelerometry. The variables were analyzed using multiple hierarchical Poisson regression. In 150 individuals (men, 14.67%; and women, 85.33%), with a mean age of 39.6 ± 0.7 years, there was a high prevalence of pain (89.33%), severe pain (69.33%), and pain in four or more regions (53.33%). The associated factors were shorter MVPA time with pain (*p* = 0.010); arthritis/arthrosis (*p* = 0.007) and the use of muscle relaxants (*p* = 0.026) with severe pain; and economic class C (*p* = 0.033), and economic class D (*p* = 0.003), along with arthritis and arthrosis (*p* = 0.025) with pain in four or more sites. There were no significant associations between sedentary behavior and any of the three outcomes analyzed. These findings indicate that, in severely obese individuals, shorter MVPA time is associated with a higher prevalence of pain. Future studies on physical activity intervention may contribute to the reduction in the prevalence and severity of pain in adults with severe obesity.

## 1. Introduction

The worldwide prevalence of obesity (body mass index, BMI ≥ 30 kg/m^2^) and severe obesity (BMI ≥ 35 kg/m^2^) has increased considerably in the last 40 years [1,2], with a higher prevalence in women [3]. A high BMI, as is seen in obese and severely obese individuals, represents a risk factor for many chronic health problems, including musculoskeletal diseases (e.g., osteoarthritis, low back pain, osteoporosis, gout, and fibromyalgia), which are considered to be the second leading cause of years spent with disability [3].

Obese adults have a high prevalence of pain, in general, and severe pain, particularly in a variety of areas such as the lower back, knee, feet, soft tissues, bones, and joints [4,5,6]. In addition, obese individuals with musculoskeletal pain have lower levels of physical activity [4,7]. The relationship between obesity and musculoskeletal pain is multifactorial, and many studies investigating pain-associated factors have been conducted in adults and the elderly [8,9,10,11,12]. The available data on pain in the obese show that low back pain is associated with reduced sleep, poor quality of life, nocturia, divorce [4], smoking, time spent sedentary, and low physical activity [13]. In obese adults, pain in more than two sites was more frequent in women and individuals in the higher BMI ranges. In addition, low levels of physical activity and sedentary behavior are considered to be potential predictors for both obesity and higher pain reporting [3,13]. The coexistence of pain and obesity often leads to a vicious cycle of pain [14], which may be influenced by physical activity [7].

There are a few studies in the literature regarding severe obesity and their associated factors such as physical activity practice and sedentary behavior [4,7,13,15]. Therefore, it is important to explore whether there are associations among severe obesity, pain, and physical activity/sedentary behavior. Such studies may provide a better understanding of these conditions, thus enabling more effective pain prevention and treatment alternatives. In this context, the aim of this study was to assess the prevalence of pain, severe pain, and widespread localization of pain in four or more sites associated with physical activity and sedentary behavior in severely obese adults, as well as to investigate any potential impact of sociodemographic status, lifestyle, and food intake, along with clinical and anthropometric variables.

## 2. Methods

### 2.1. Study Design

This study presents the baseline data of the clinical trial “DieTBra Trial,” registered on the ClinicalTrials.gov platform (NCT02463435) [16,17,18]. The data collection was conducted between June 2015 and September 2016. This study was approved by the Research Ethics Committee (CEP/HC/UFG) under protocol number 747.792/2014; all subjects signed the Informed Consent Form. The DieTBra Trial evaluates several outcomes [19,20,21,22].

### 2.2. Participants

Adults aged between 18 and 65 years, of both genders, who were severely obese (with BMI ≥ 35 kg/m^2^) were included in the study, after being referred to our outpatient clinic by the primary care department of the Brazilian Public Healthcare System (SUS—Sistema Único de Saúde). Pregnant women, nursing mothers, people with disabilities who were unable to walk, who had bariatric surgery, or who had been receiving nutritional treatment or dietary regulation for weight loss in the previous 2 years were excluded, as were those who were currently taking antiobesity or anti-inflammatory medication, or were diagnosed with chronic obstructive pulmonary disease or cancer.

### 2.3. Measures

#### 2.3.1. Independent Variables

##### Sociodemographic Data, Lifestyle, and Food Consumption

Sociodemographic, lifestyle, and food consumption data were collected using a standardized questionnaire that was previously tested. The sociodemographic variables were: gender, age, marital status (living with or without a partner), occupation (formal, informal, self-employed, retired, and housewife), head of household’s level of education, and economic class based on the Economic Classification Criterion for Brazil of the Brazilian Association of Research Companies—ABEP (sum of household items plus education of the head of household) [23].

Lifestyle variables were: smoking (if the patient smokes or has ever smoked a cigarette/pipe/cigar at any time), binge drinking (referring to the consumption of five or more units of any kind of beverage containing alcohol in a single occasion for males and four or more units for females) assessed by the simplified version of the study entitled Gender, Alcohol and Culture: an International Study—GENACIS [24,25]. Physical activity and sedentary behavior were assessed using the accelerometer Triaxial ActiGraph wGT3X (ActiGraph, Pensacola, FL, USA). Accelerometers were used on the nondominant wrist (above the ulnar styloid process), and subjects were instructed to wear them 24 h a day, including while bathing and sleeping, for 6 consecutive days. The accelerometer sampling frequency was set to 30 Hz, and the data collection interval was set to a 1 min *Epoch*. The accelerometers were configured, and the respective data were accessed using the software ActiLife 6.11.7. The output data were processed using the R GGIR package (http://cran.r-project.org). The outcome measure used in this study was moderate to vigorous physical activity (MVPA), which was analyzed using a 5-min bout. Nonbouted light physical activity (LPA) represented more routine activities, while a 5-min MVPA bout represented more structured activities [26,27]. The sedentary time (<50 mg, without bouts) was measured in min per day [28]. The variables MVPA, LPA, and sedentary behavior (SB) were categorized using the median values. The cutoffs for classifications were: for the SB time: ˂1182.15 min/day as lower SB and ≥1182.15 min/day as higher SB; for LPA: ˂164.89 min/day as lower LPA time and ≥164.89 min/day as higher LPA; and for MVPA: ˂8.48 min/day as lower MVPA and ≥8.48 min/day as higher MVPA.

The food consumption variables were restricted to the consumption of fruits and vegetables. Daily intake of boiled vegetables (such as squash, okra, chayote, cauliflower, and broccoli) and fresh fruits was assessed using the Food Frequency Questionnaire (FFQ) [29].

##### Clinical Variables

The clinical variables assessed were: anxiety, depression, arthritis/arthrosis, type 2 diabetes, fall experienced in the last 12 months, bone fractures, medications taken, and biochemical tests of blood samples. For anxiety and depression, the Hospital Anxiety and Depression Scale was applied [30]. For the other variables, the following questions were asked: “Have you ever had a fracture in your life?”; “Have you had a fall in the last 12 months?”; “Indicate if your doctor has already told you that you have any of the following conditions: arthritis/arthrosis/joint problems?”. The diagnosis of type 2 diabetes was based on the criteria of the American Diabetes Association, which includes concentrations of fasting glucose ≥126 mg/dL, glycated hemoglobin ≥6.5%, and/or use of hypoglycemic agents [31].

The drug products analyzed included analgesics, nonsteroidal anti-inflammatory drugs (NSAIDs), statins, and muscle relaxants. These were of continuous use and were classified according to the Anatomical Therapeutic Chemical Classification System (ATC codes) according to their mechanisms of action [32].

Blood was collected after 12 h of fasting for biochemical tests to quantify uric acid (mg/dL), C-reactive protein (CRP; mg/dL), vitamin D (ng/mL), glucose (ng/mL), and glycated hemoglobin (%). The reference values were: male uric acid 2.5–7.0 mg/dL and female uric acid 1.5–6 mg/dL [33]; CRP reagent >6 mg/L. Vitamin D deficiency was considered if the serum concentration of vitamin D was below 20 ng/mL, insufficiency if it was between 21 and 29 ng/mL, and sufficiency if it was over 30 ng/mL [34].

##### Anthropometry

The information on body weight and height was used to calculate the BMI (BMI = weight (kg)/height (m)^2^). Body weight was measured on a digital platform scale capable of weighing up to 200 kg within 100 g of accuracy (Welmy, USA). For height measurements, we used a stadiometer attached to a digital scale, which provides accuracy within 0.1 cm. The calculation and classification of BMI followed the World Health Organization recommendations [35]: severe obesity: 35–39.9 kg/m^2^; morbid obesity 40–49.9 kg/m^2^; and super obesity ≥50.0 kg/m^2^.

#### 2.3.2. Outcome Variables

##### Musculoskeletal Pain

The Nordic Musculoskeletal Questionnaire was applied to measure the musculoskeletal pain [36,37,38]. This instrument has been validated and adapted to the Brazilian population for the purpose of estimating the prevalence of pain [39]. Outcomes based on pain symptoms were reported for nine anatomical regions over the previous 7 days: neck, shoulders, elbows, upper back, lower back, wrist/hands, hip/thighs, knees, and ankles/feet [36,37,40]. Three outcomes were considered: pain (yes/no), severe pain (yes/no), and pain in four or more sites (yes/no). Severe pain was assessed using the Numerical Pain Rating Scale [41], which classifies a score of eight or higher as severe pain. Severe pain was classified according to the criteria of Boonstra et al. (2016), which considers: no pain (score = 0), mild pain (≤5), moderate pain (6 and 7), and severe pain (≥8) [42].

### 2.4. Statistical Analysis

The database was built using the program EPI DATA^®^ version 3.1; two people entered and verified the information. For the analyses, the statistical package Stata version 13.0 (Stata Corp LP, College Station, TX, USA) was used. Statistical significance was established using a cutoff value of *p* < 0.05.

The Kolmogorov–Smirnov test was used to assess normality for physical activity and sedentary behavior variables. For normally distributed variables (sedentary behavior and LPA), the Student’s *t*-test was used; for the variables that were not normally distributed, the Mann-Whitney U test was used for comparison between MVPA and presence of pain in general, presence of severe pain, and pain in four or more regions.

Descriptive analyses are presented in absolute numbers (*n*) and relative frequencies (%), along with the mean and standard deviation. The Chi-square test (χ^2^) or Fisher’s exact test were used in the bivariate analysis. Poisson regression was used to calculate the prevalence ratio and 95% confidence interval, whereas the *p*-value was obtained using the Wald test. Variables with *p*-value < 0.20 in the bivariate analysis were included in multiple hierarchical Poisson regression analyses, with robust variance based on a hierarchical model [43]. The independent variables in this hierarchical analysis were grouped into three categories: (I) demographic data (gender, education, and economic class; (II) diet and exercise (fruit and vegetable consumption and MVPA [min/day]); and (III) clinical characteristics (falls in the last 12 months, fracture, anxiety, depression, arthritis/arthrosis, use of analgesics, and muscle relaxant use). In the multivariate analysis, variables without statistical power were excluded (n < 10 in all of the strata) [44].

## 3. Results

**Figure 1:**
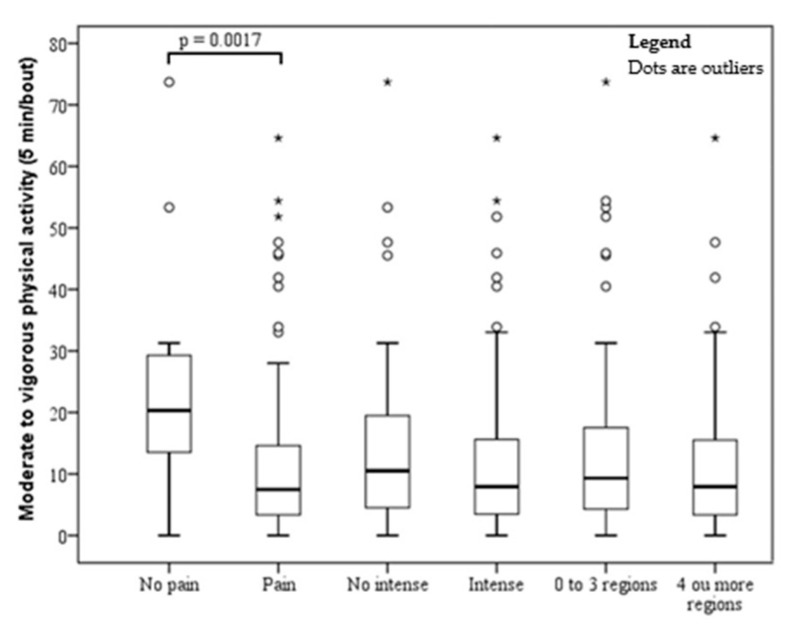
Boxplot of time spent on moderate to vigorous physical activity by obese patients without and with pain, intensity, and four or more painful sites. *°outliers

**Figure 2:**
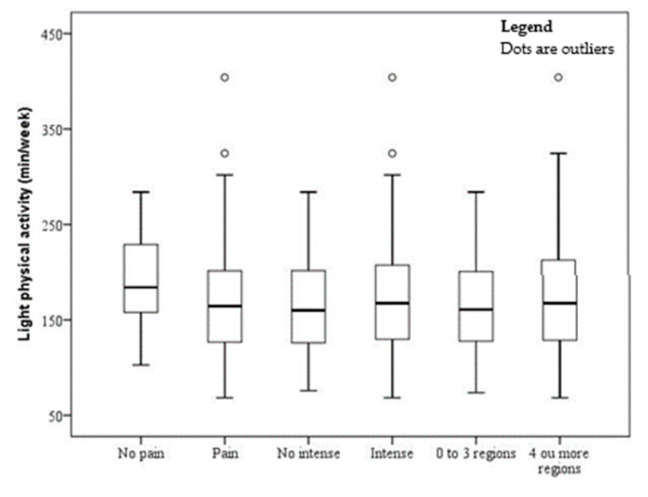
Boxplot of time spent on light physical activity by obese patients without and with pain, intensity, and four or more painful sites. °outliers

**Figure 3:**
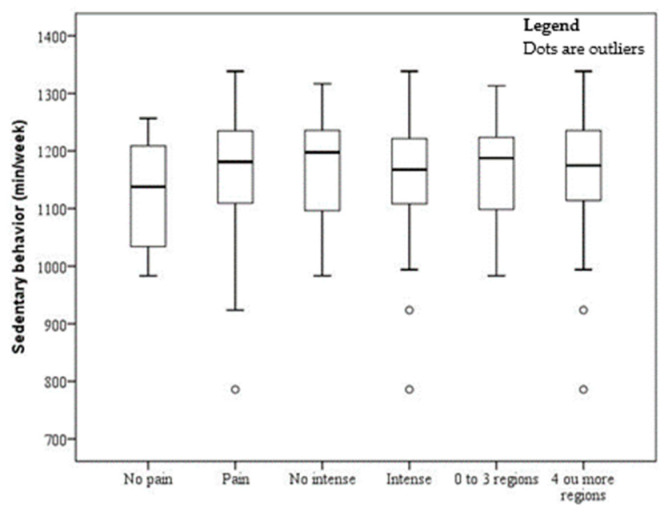
Boxplot of time spent in sedentary behavior by obese patients without and with pain, intensity, and four or more painful sites. °outliers

The sample comprised 150 subjects (men, 14.67% and women, 85.33%), with a mean age of 39.57 ± 0.72 years and a mean BMI of 46.12 ± 0.53 kg/m^2^. The prevalence of musculoskeletal pain, severe pain, and pain in four or more sites were 89.33%, 69.33%, and 53.33%, respectively. The mean time of sedentary behavior was 1167.65 ± 89.82 (min/day), LPA was 170.55 ± 55.54 (min/day), and MVPA was 12.74 ± 13.70 (min/day). The associations of MVPA, LPA, and sedentary behavior with pain, severe pain, and number of painful sites are shown in Figure 1, Figure 2 and Figure 3. There was a significant association between pain and MVPA (*p* = 0.0017), indicating that individuals with shorter MVPA have more pain (Figure 1). There was no significant association between pain and LPA (Figure 2) and between pain and sedentary behavior (Figure 3).

The characteristics of the population studied in terms of pain, severe pain, and the number of painful sites are presented in Table 1 and Table 2. Bivariate analysis indicated that pain was associated with shorter MVPA time and muscle relaxant use; severe pain with fracture, arthritis/arthrosis, and muscle relaxant use; and pain in four or more sites with females, low economic class, anxiety, depression, and arthritis/arthrosis (Table 1 and Table 2).

Variables with *p*-values less than 0.20 were included in the multivariate analysis. For pain in general, these included gender, fruit consumption, MVPA, anxiety, and use of muscle relaxant; for severe pain, schooling, economic class, fall in the last 12 months, fracture, anxiety, depression, arthritis/arthrosis, and use of muscle relaxant; and for pain in four or more sites, economic class, anxiety, depression, arthritis/arthrosis, use of analgesics, and muscle relaxant use.

Following the multiple analysis, presence of pain was still associated with lower MVPA (*p* = 0.010), severe pain was associated with the presence of arthritis/arthrosis (*p* = 0.007) and the use of muscle relaxants (*p* = 0.026), and the presence of pain in four or more sites was associated with low economic class (C (*p* = 0.033) and D (*p* = 0.003)) and arthritis/arthrosis (*p* = 0.025) (Table 3).

## 4. Discussion

To the best of our knowledge, this is the first study to investigate the prevalence and associated factors of pain, severe pain, and pain in four or more regions associated with physical activity and sedentary behavior in adults with severe obesity. Among the contributions of this article, we highlight the following results in severely obese adults: high prevalence of pain, severe pain, and pain in four or more regions; low MVPA time and elevated sedentary behavior time; an association between MVPA and higher prevalence of musculoskeletal pain; severe pain associated with arthritis/arthrosis and the use of muscle relaxants; and pain in four or more sites associated with low economic class and arthritis/arthrosis. These results are important in the field of clinical research and could contribute in the clinical contexts related to the treatment of severe obesity and pain, as well as pain prevention in severely obese adults to provide a greater incentive for participating in physical activity; this is an easily addressable risk factor that could benefit severely obese individuals.

Our findings regarding the high prevalence of pain, severe pain, and pain in four or more regions in adults with severe obesity are consistent with other studies in the same patient populations [4,5,13,15,45,46]. In Brazil, the prevalence of pain in adults with BMI ≥ 35 kg/m^2^ ranges from 66.7% to 90.1% [46,47]. The prevalence of pain in several body regions has also been reported in many studies [4,5,48]. Our results indicate that pain in adults with severe obesity cannot be neglected, and its treatment should occur concurrently with obesity treatment.

In the present study, individuals with a shorter MVPA time and a longer duration of sedentary behavior experienced more pain. Despite the differences in the origins and pathogeneses in most individuals, the pain is characterized by deficient physical function, mobility limitations, depression, anxiety, and sleeping disorders. Physical activity promotes an improvement in all these parameters, and any level of physical activity is preferable to sedentary behavior [49]. Obesity can contribute to musculoskeletal pain and, consequently, it also contributes to an insufficient amount of MVPA along with increased sedentary behavior [14,50,51]. Although no studies regarding adults with obesity were found, in studies on nonobese adults and the elderly, chronic musculoskeletal pain and a higher number of painful sites were associated with low MVPA [52,53,54]. The interdisciplinary treatment of patients with severe obesity is critical, especially those in pain. Patients with severe obesity who underwent treatment through the Interdisciplinary Multimodal Pain Rehabilitation program showed improvement in physical and mental conditions and maintained these benefits at a 12-month follow-up [55]. Improvement in the physical activity in the severely obese enables better adherence to the practice of physical activity that is often not achieved due to difficulty in staying active and motivated [52,53,54].

Severe pain and pain in four or more sites were associated with arthritis/arthrosis. These findings are consistent with previous studies that have shown that obese adults have a high prevalence of arthritis, which is often associated with severe pain intensity and widespread pain in several regions [56,57,58]. Weight loss treatment has been reported to have positive effects on individuals with arthritis and obesity [58]. In addition, individuals with rheumatoid arthritis should be encouraged to exercise as part of their routine care [59]. Therefore, educational interventions involving self-management should be performed to reduce pain and improve physical function and quality of life. Further, physical training should be included in the treatment of obese adults with arthritis [57,59]. Physical training for individuals with severe obesity can involve a fitness program that includes a warm-up, stretching, and resistance training phase [60].

There was an association between severe pain and muscle relaxant use. Muscle relaxants provide clinically significant relief from acute pain, particularly in the lower back [61], and should be used temporarily for pain relief [62]. The efficacy of muscle relaxants for chronic pain is unknown [61]. Although we have not found evidence for the cause of the association between severe pain and the use of muscle relaxants, we believe that the high prevalence of pain and the use of this medication in higher quantities than recommended may have contributed to this result [62]. However, this is an important finding and serves as a warning for consistent use of muscle relaxants and possible adverse effects.

Pain in four or more regions was associated with low economic class. This finding is consistent with previous research on adults, with and without obesity [63,64,65,66,67,68]. Studies have shown that low economic class and financial concerns are associated with severe pain and widespread pain in several body regions [63,64,65,66,67,68]. In contrast, those with higher incomes tend to have a healthier life, live longer, and suffer less from illness and disability [69,70,71].

The strengths of this investigation are the inclusion of individuals with severe obesity and the rigorous controls used at all stages of the research. The research team was well trained, and the rules and procedures were strictly applied. Another relevant aspect of this study is the assessment of pain and the analysis of several variables that had not been investigated in previous studies. The possible limitations of this study are related to memory bias associated with self-reporting as a result of using the Nordic Musculoskeletal Questionnaire and the Numerical Pain Rating Scale. However, to minimize the impact of the bias, we opted to use the pain scores reported during the previous 7 days. Other limitations were the nonuse of the cutoff points established in the literature for determining the level of physical activity and sedentary behavior due to the characteristics of the population assessed (low levels of physical activity and prolonged sedentary behavior). These last two limitations were mitigated by using the median to establish the cutoff point. Another limitation of this study is the low number of men in our study sample. However, it should be noted that women seek treatment for obesity and bariatric surgery more frequently than men both in Brazil and in the United States [72]. In addition, this is a study on severely obese individuals whose prevalence in Brazil is low but growing. Thus, the sample size is representative of this population, and these data are critical in stimulating the development of clinical treatments and preventing other comorbidities and pain in these individuals.

## 5. Conclusions

This study provides evidence of the high prevalence of pain, severe pain, and pain in four or more regions in severely obese adults. In addition, this study is the first to demonstrate the factors associated with pain and their characteristics in severely obese individuals. We emphasize the importance of moderate to vigorous physical activity, which can contribute to the reduction of symptoms of pain, arthritis/arthrosis, and the use of muscle relaxants. Several physical activity modalities are available at a low cost for participants and are affordable for all social classes. Moderate to vigorous physical activity alone is important for both pain and obesity treatment. Future studies on physical activity intervention may contribute to the reduction in the prevalence and severity of pain in adults with severe obesity. It is crucial to establish intervention protocols to treat the pain in this group of individuals.

## Figures and Tables

**Table 1:** Prevalence and bivariate analysis of the association of pain, severe pain, and number of pain sites with the variables of socio-demographic, status, food intake, and lifestyle in severely obese adults (*n* = 150).

Variable	Pain			Severe Pain			Four or More Painful Sites		
	Prevalence *n* (%)	PR (95% CI)	*p*	Prevalence *n* (%)	PR (95% CI)	*p*	Prevalence *n* (%)	PR (95% CI)	*p*
Gender			0.158			0.901			0.036
Male	17 (77.27)	1		15 (68.18)	1		6 (27.27)	1	
Female	117 (91.41)	1.18 (0.94–1.49)		89 (69.53)	1.02 (0.75–1.39)		74 (57.81)	2.12 (1.05–4.27)	
Age (years)			0.406 *			0.687			0.864
18–39	69 (90.79)	1		51 (67.11)	1		39 (51.32)	1	
40–49	48 (90.57)	0.99 (0.89–1.12)		37 (69.81)	1.04 (0.82–1.32)		29 (54.72)	1.07 (0.77–1.48)	
50 or more	17 (80.95)	0.89 (0.72–1.11)		16 (76.19)	1.14 (0.85–1.51)		12 (57.14)	1.11 (0.72–1.71)	
Occupation			0.968			0.936			0.678
Formal worker	45 (90.00)	1.01 (0.89–1.16)		34 (68.00)	0.96 (0.74–1.24)		26 (52.00)	1.04 (0.71–1.52)	
Informal worker/self-employed	46 (88.46)	1		37 (71.15)	1		26 (50.00)	1	
Retired/housewife and others ^1^	43 (89.58)	1.01 (0.88–1.16)		33 (68.75)	0.97 (0.75–1.25)		28 (58.33)	1.17 (0.81–1.68)	
Lives with partner			0.552			0.748			0.204
No	48 (87.27)	0.96 (0.85–1.09)		39 (70.91)	1.04 (0.83–1.29)		33 (60.00)	1.21 (0.90–1.63)	
Yes	86 (90.53)	1		65 (68.42)	1		47 (49.47)	1	
Schooling (years)			0.598			*0.081*			0.744
≤10	66 (88.00)	0.97 (0.87–1.08)		57 (76.00)	1.21 (0.97–1.51)		41 (54.67)	1.05 (0.78–1.42)	
≥11	68 (90.67)	1		47 (62.67)	1		39 (52.00)	1	
Economic class			0.303 *			0.079 *			0.004
A-B	28 (82.35)	1		19 (55.88)	1		10 (29.41)	1	
C	83 (90.22)	1.09 (0.92–1.30)		65 (70.65)	1.26 (0.91–1.75)		52 (56.52)	1.92 (1.11–3.34)	
D	23 (95.83)	1.16 (0.97–1.39)		20 (83.33)	1.49 (1.05–2.11)		18 (75.00)	2.55 (1.44–4.52)	
Smoker			0.267 *			0.232			0.508
No	88 (87.13)	1		67 (66.34)	1		52 (51.49)	1	
Yes	46 (93.88)	1.08 (0.97–1.20)		37 (75.51)	1.14 (0.92–1.41)		28 (57.14)	1.11 (0.82–1.51)	
Alcohol consumption			0.450			0.497			0.981
No	97 (90.65)	1.05 (0.92–1.21)		76 (71.03)	1.09 (0.85–1.40)		57 (53.27)	0.99 (0.71–1.39)	
Yes	37 (86.05)	1		28 (65.12)	1		23 (53.49)	1	
Fruit consumption (daily)			0.173			0.774			0.528
No	101 (91.82)	1.11 (0.95–1.30)		77 (70.00)	1.04 (0.81–1.33)		57 (51.82)	0.90 (0.65–1.24)	
Yes	33 (82.50)	1		27 (67.50)	1		23 (57.50)	1	
Vegetable consumption (daily)			1.000 *			0.867			0.420
No	97 (88.99)	1		76 (69.72)	1		56 (51.38)	1	
Yes	37 (90.24)	1.01 (0.90–1.14)		28 (68.29)	0.98 (0.77–1.25)		24 (58.54)	1.14 (0.83–1.56)	
Sedentary time (min/day)			0.399			0.432			0.680
<Median (1182.15)	63 (88.73)	0.95 (0.86–1.06)		52 (73.24)	1.09 (0.88–1.35)		40 (56.34)	1.06 (0.79–1.44)	
≥Median (1182.15)	65 (92.86)	1		47 (67.14)	1		37 (52.86)	1	
LPA (min/day)			0.752			0.498			0.551
<Median (164.89)	65 (91.55)	1		48 (67.61)	1		37 (52.11)	1	
≥Median (164.89)	63 (90.00)	0.98 (0.88–1.09)		51 (72.86)	1.08 (0.87–1.34)		40 (57.14)	1.10 (0.81–1.48)	
MVPA (min/day)			0.009 *			0.432			0.680
<Median (8.48)	69 (97.18)	1.15 (1.03–1.29)		52 (73.24)	1.09 (0.88–1.35)		40 (56.34)	1.06 (0.79–1.44)	
≥Median (8.48)	59 (84.29)	1		47 (67.14)	1		37 (52.86)	1	

^1^ Other includes: student, rural worker, unemployed, laid off, receiving pension, and sick-pay recipient. CI: confidence interval; LPA: light physical activity; MVPA: moderate to vigorous physical activity; PR: adjusted prevalence ratio. Wald test was used to calculate all “*p*” values, except when frequencies were below five, in which case, Fisher’s exact test * was used. *p* < 0.05 was considered statistically significant (bold mark). Variables with *p* < 0.20 were further analyzed by multiple hierarchical Poisson regression.

**Table 2:** Prevalence and bivariate analysis of the association of pain, severe pain, and number of pain sites with the clinical and anthropometric variables in severely obese adults (*n* = 150).

Variable	Pain			Severe Pain			Four or More Painful Sites		
	Prevalence *n* (%)	PR (95% CI)	*p*	Prevalence *n* (%)	PR (95% CI)	*p*	Prevalence *n* (%)	PR (95% CI)	*p*
Fall in the last 12 months			0.354 *			0.155			0.219
No	84 (86.60)	1		64 (65.98)	1		51 (52.58)	1	
Yes	34 (94.44)	1.09 (0.97–1.22)		28 (77.78)	1.18 (0.94–1.48)		23 (63.89)	1.22 (0.89–1.66)	
Fracture			0.554 *			0.028			0.249
No	83 (87.37)	1		61 (64.21)	1		50 (52.63)	1	
Yes	35 (92.11)	1.05 (0.93–1.19)		31 (81.58)	1.27 (1.03–1.57)		24 (63.16)	1.2 (0.88–1.64)	
Anxiety			0.188			0.060			0.006
No	34 (82.93)	1		23 (56.10)	1		13 (31.71)	1	
Yes	100 (91.74)	1.11 (0.95–1.29)		81 (74.31)	1.32 (0.99–1.78)		67 (61.47)	1.94 (1.21–3.12)	
Depression			0.279			0.080			0.002
No	47 (85.45)	1		33 (60.00)	1		19 (34.55)	1	
Yes	87 (91.58)	1.07 (0.95–1.21)		71 (74.74)	1.25 (0.97–1.59)		61 (64.21)	1.86 (1.25–2.76)	
Arthritis/arthrosis			0.524 *			0.002 *			0.007
No	104 (88.14)	1		75 (63.56)	1		57 (48.31)	1	
Yes	30 (93.75)	1.06 (0.95–1.19)		29 (90.63)	1.43 (1.19–1.70)		23 (71.88)	1.49 (1.12–1.98)	
Type 2 diabetes			0.828			0.610			0.501
No	80 (88.89)	1		61 (67.78)	1		46 (51.11)	1	
Yes	54 (90.00)	1.01 (0.90–1.13)		43 (71.67)	1.06 (0.85–1.31)		34 (56.67)	1.11 (0.82–1.50)	
Use of analgesics			0.615			0.932			0.113
No	76 (90.48)	1		58 (69.05)	1		40 (47.62)	1	
Yes	58 (87.88)	0.97 (0.87–1.09)		46 (69.70)	1.01 (0.81–1.25)		40 (60.61)	1.27 (0.94–1.71)	
Use of anti-inflammatory agents			0.362 *			0.754			0.597
No	101 (87.83)	1		79 (68.70)	1		60 (52.17)	1	
Yes	33 (94.29)	1.07 (0.97–1.19)		25 (71.43)	1.04 (0.81–1.33)		20 (57.14)	1.10 (0.78–1.53)	
Use of statin			-			0.724 *			1.000*
No	124 (88.57)	-		96 (68.57)	1		75 (53.57)	1	
Yes	10 (100.00)	-		8 (80.00)	1.17 (0.84–1.62)		5 (50.00)	0.93 (0.49–1.77)	
Use of muscle relaxant			0.016 *			0.037			0.291
No	56 (82.35)	1		41 (60.29)	1		33 (48.53)	1	
Yes	78 (95.12)	1.16 (1.02–1.30)		63 (76.83)	1.27 (1.02–1.60)		47 (57.32)	1.18 (0.87–1.61)	
Uric acid (mg/dL)			0.430 *			0.640			0.344
Normal	118 (90.08)	1.07 (0.87–1.31)		90 (68.70)	0.93 (0.69–1.25)		72 (54.96)	1.31 (0.75–2.27)	
High	16 (84.21)	1		14 (73.68)	1		8 (42.11)	1	
C-reactive protein (mg/dL)			0.418			0.963			0.885
Nonreactive	98 (90.74)	1.06 (0.92–1.22)		75 (69.44)	1.01 (0.79–1.28)		58 (53.70)	1.03 (0.73–1.44)	
Reactive	36 (85.71)	1		29 (69.05)	1		22 (52.38)	1	
Vitamin D (ng/mL)			0.450 *			0.467			0.594
Deficiency	28 (96.55)	1		17 (58.62)	1		14 (48.28)	1	
Insufficiency	45 (88.24)	0.91 (0.81–1.03)		37 (72.55)	1.24 (0.87–1.76)		30 (58.82)	1.22 (0.78–1.90)	
Sufficiency	61 (87.14)	0.90 (0.81–1.01)		50 (71.43)	1.22 (0.87–1.71)		36 (51.43)	1.07 (0.68–1.65)	
Degree of obesity (kg/m^2^)			0.668 *			0.539			0.585
35–39.9	23 (92.00)	1		15 (60.00)	1		12 (48.00)	1	
40–49.9	76 (90.48)	0.98 (0.86–1.13)		61 (72.62)	1.21 (0.85–1.71)		48 (57.14)	1.19 (0.76–1.87)	
≥50	35 (85.37)	0.93 (0.78–1.10)		28 (68.29)	1.14 (0.77–1.67)		20 (48.78)	1.02 (0.61–1.70)	

CI: confidence interval; PR: adjusted prevalence ratio. Wald test was used to calculate all “*p*” values, except when frequencies were below five, in which case, the Fisher’s exact test was used (indicated by *). *p* < 0.05 was considered statistically significant (bold mark). Variables with *p* < 0.20 were further analyzed by multiple hierarchical Poisson regression.

**Table 3:** Multiple analysis of the association of pain, severe pain, and four or more painful sites with the independent variables in severely obese adults.

Variable	Pain	Severe Pain	Four or More Painful Sites	Variable	Pain	
	PR (95% CI)	*p*	PR (95% CI)	*p*	PR (95% CI)	*p*
First Level						
Gender *			–	–	–	–
Male	1		–		–	
Female	1.21 (0.95–1.54)	0.127	–		–	
Schooling (years)	–	–			–	–
≤10	–		1.09 (0.87–1.36)	0.478	–	
≥11	–		1		–	
Economic class	–	–				
A–B	–		1		1	
C	–		1.15 (0.83–1.59)	0.417	1.71 (1.05–2.81)	**0.033** ^a^
D	–		1.29 (0.89–1.88)	0.176	2.15 (1.30–3.58)	**0.003** ^a^
Second Level						
Fruit Consumption (daily)			–	–	–	–
No	1.13 (0.97–1.31)	0.109	–		–	
Yes	1		–		–	
MVPA (min/day) (median)			–	–	–	–
<Median (8.48)	1.15 (1.03–1.28)	**0.010** ^a^	–		–	
≥Median (8.48)	1		–		–	
Third Level						
Fall in the last 12 months	–	–			–	–
No	–		1		–	
Yes	–		1.06 (0.84–1.33)	0.621	–	
Fracture	–	–			–	–
No	–		1		–	
Yes	–		1.23 (0.99–1.54)	0.068	–	
Anxiety						
No	1		1		1	
Yes	1.07 (0.93–1.23)	0.337	1.17 (0.84–1.62)	0.358	1.45 (0.86–2.45)	0.159
Depression	–	–				
No	–		1		1	
Yes	–		1.03 (0.79–1.36)	0.810	1.42 (0.94–2.15)	0.096
Arthritis/arthrosis	–	–				
No	–		1		1	
Yes	–		1.31 (1.08–1.59)	**0.007** ^a^	1.38 (1.04–1.82)	**0.025** ^a^
Use of analgesics	–	–	–	–		
No	–		–		1	
Yes	–		–		1.30 (0.99–1.71)	0.059
Use of muscle relaxant					–	–
No	1		1		–	
Yes	1.07 (0.96–1.19)	0.211	1.31 (1.03–1.66)	**0.026** ^a^	–	

CI: confidence interval; PR: prevalence ratio. * The gender variable was excluded from the multiple analysis, only for pain in four or more sites. Wald test was used to calculate all *p*-values. ^a^ *p* < 0.05 was considered statistically significant (bold mark).
